# The Maxillary Sinus—Chronic Empyema Thereof; Its Treatment

**Published:** 1899-04

**Authors:** J. A. Stucky

**Affiliations:** Lexington, Ky.


### The Maxillary Sinus—Chronic Empyema Thereof; Its Treatment.

BY J. A. STUCKY, M.D., LEXINGTON, KY.

Presented to the Section on Laryngology and Otology, at the Forty-ninth Annual Meeting of the American Medical Association, held at Denver, Colorado, June 7-10, 1898.

So much has been written upon this subject that it may seem superfluous at this time to again bring it before you for consideration. That empyema of the nasal accessory cavities, especially the antrum of Highmore, is far more frequent than is generally supposed, and as frequently overlooked, I think "may go without saying." Failure to relieve many of our so-called "old catarrhal cases" after we have removed all visible obstructive and diseased tissue in the nasal and pharyngo-nasal cavities, is due to not recognizing suppurative and other pathologic conditions in the accessory cavities. Empyema of the maxillary sinus is caused most frequently by dental caries, and the removal of a dead tooth that penetrates the cavity usually gives prompt relief, unless the suppurative process has continued long enough to result in caries and granular or polypoid degeneration of the lining intra-cellular membrane.

The cause which interest us most is that which comes from the nose, or infection in the frontal or ethmoidal sinus. Atrophic rhinitis or ozena may be a possible cause of suppuration of the antrum by infection or infiltration through the ostium maxillare. In five of my cases where marked atrophic trouble existed, with shrunken and degenerated (polypoid) middle turbinate, with no dental caries or evidence of suppuration in any of the other accessory cavities, the antrum was full of pus and granulations.

In these cases I am at a loss to know to what to attribute the empyemic condition, unless it be the infection from the muco-purulent and purulent secretion from the nasal cavities. Another probable cause is the damming up of the ostium maxillare by nasal polypi or enlarged middle turbinate, the retained secretion undergoing such a change as to result in irritation, inflammation and suppuration.

The symptoms of this trouble are frequently undefined and misleading. Very little attention is drawn by the patient to the cheek. The pain most complained of is in the back of the head and the supra-orbital and temporal region. I need not emphasize the necessity of a very careful examination in order to make an accurate diagnosis. Tapping sharply with a heavy probe the first, the second, and the cuspid teeth, will cause pain if the suppuration is extensive or if there is dental caries. Trans-illumination is frequently a great aid, but is by no means certain. When in doubt, the exploratory puncture with trocar and canula or small burr attached to dental engine will settle the question beyond a doubt.

In the treatment of these cases our first attention should be directed to the nasal cavity. If there be any sobtruction to free drainage or respiration, it should be removed. If the middle turbinate is so enlarged as to encroach upon the middle meatus, or enough to interfere with the ostium maxillare, the anterior portion should be removed. In the majority of cases I have found this operation of turbinectomy to be of greatest value. If the ethmoid cells are involved in the suppurative process or have undergone a polypoid degeneration, these should be thoroughly dealt with, and the same may be said of the frontal sinus. Sometimes we find the maxillary sinus filled with pus and granulations, with no symptom of frontal or ethmoidal disease, but a close examination often reveals trouble in one or both of these—more frequently the ethmoid.

Having attended to the nasal cavity, the operation upon the maxillary sinus is performed in the following manner: If necessary the second molar is extracted, and with Buck's mastoid drill a large opening is made through the alveolar process into the antrum. With a sharp-edged bone spoon the opening is made smooth, and shaped to admit the introduction of a medium or large-sized speculum, so the cavity can be illuminated (with head mirror) and thoroughly examined. If granulations, polypi, necrosis, or caries exist, Myles' curettes are used to remove all diseased tissue. After this is done the cavity is thoroughly irrigated and loosely packed with iodoform gauze. The first dressing is not removed for forty-eight hours, unless elevation of temperature and throbbing pain indicate the necessity for so doing. After the removal of the first dressing the antrum should be irrigated daily, the opening and drainage maintained by a tampon of sterilized or iodoform gauze. I think that in many cases, where a large opening is made, and the cavity thoroughly cleaned and drained, spontaneous healing takes place "by the filling up of the antrum with connective tissue." The nasal cavities should be cleaned with alkaline antiseptic spray, anteriorly and posteriorly.

Of the cases which I reported at the meeting of the Kentucky Medical Society in 1896,* all remain free from antral trouble, and I desire to emphasize the superiority of entering the antrum through the alveolar process, rather than the cuspid fossa. By the latter procedure, in order to make an entrance sufficiently large, we are liable to split the thin, bony (external) wall, leading to prolonged and painful complications. The tendency of the soft parts of the cheek to overlap and close the opening seriously interferes with drainage and is a source of constant annoyance and irritation to the patient. A counter-opening through the inferior meatus to facilitate drainage, I have never found necessary, and do not think it advisable. The gauze drainage has proved far more satisfactory to me than either metal or rubber tubes.

DISCUSSION.

Dr. MELVILLE BLACK: I desire to endorse two points made by Dr. Stucky: the place of making the opening into the antrum, and the non-use of tubes. The loss of the tooth is a small thing to be considered when a cure is to be accomplished in a disease so essentially chronic and hard to cure. By making the

* American Practitioner and News, June 27, 1896.

opening through the alveolar process, a hole can be made large enough to remain open a long time. The tube causes granulation tissue formation about the entrance of the tube into the antrum. This end of the tube soon becomes covered with granulations, repeated curettement being required to prevent it.

I am surprised that Dr. Stucky has not laid more stress upon the extreme chronicity of this disease. My experience has been that it is a very hard disease to cure, that the patient becomes discouraged long before he is cured, and is liable to drift into other hands.

The tendency to granulation tissue formation after curettement is marked. A few days after curettement it has been my practice to cauterize the interior of the antrum with pure trichloracetic acid; this will prevent granulation tissue formation and hasten resolution. To apply the acid the interior of the antrum should first be cocainized; a piece of cotton is wound tightly on the end of a cotton carrier so that we will have a round ball of cotton on the end of the probe, which is about one-fourth inch in diameter. This is dipped into the acid, when it is carried into the antrum and the latter wiped over thoroughly with it. The membrane caused by the cauterization is exfoliated in about ten days. No reaction follows the application of this acid. Another method I have practiced to avoid granulation tissue formation is that of packing the antrum with strips of linen or muslin one-fourth inch wide, saturated with balsam Peru.

I desire to call attention to a method I have of opening the antrum for acute empyema: A small trephine, propelled with a dental engine, is passed into the antrum through the palatine side of the alveolar process. Drainage and cleansing is usually all that is required; it is not desirable to sacrifice a tooth, and this opening will remain patent long enough to accomplish this.

Dr. Emil Mayer: Operations for the drainage of antral abscess, made either through the alveolar process or anterior to the malar ridge, are the usual methods employed. Drainage is quite as effectual, and less annoying to the patient, when the operation is done posterior to the malar ridge. Regarding puncture, we must bear in mind that puncture if made should be performed properly, and that the thinness of the bone at the proper spot makes such procedure easy of performance. The point I enter is about one and one-fourth inches from the external orifice. Many of these antral abscesses are associated with ethmoidal disease, and here I use, after curetting, the method of washing out the cavity by attaching the silver tube to the ordinary antitoxin syringe. It has been very effective in my hands and is very convenient.

Dr. Gradle: I would like to ask Dr. Stucky what length of time was required for a cure in the different patients, and what percentage of failures he has had?

Dr. Randall said more stress might be laid upon the not infrequent occurrence of empyema of the maxillary sinus as a mere result of drainage of the frontal or ethmoid cells in to a healthy or secondarily involved Highmore. No amount of treatment of the lower cavity will cure the condition. As to the point of opening, if man were as upright as supposed, an opening directed downward would be best; but in point in fact we find that other influences, such as capillarity and the suction effect of air currents through the nose, surpass the force of gravity in determining the easiest direction of drainage.—*Am. Med. Ass'n Journal.*

